# The High Five: Associations of the Five Positive Factors with the Big Five and Well-being

**DOI:** 10.3389/fpsyg.2017.01250

**Published:** 2017-07-25

**Authors:** Alejandro C. Cosentino, Alejandro Castro Solano

**Affiliations:** ^1^Departamento de Psicología, Facultad de Ciencias Sociales, Universidad de Palermo Buenos Aires, Argentina; ^2^Facultad de Psicología, Universidad de Buenos Aires Buenos Aires, Argentina; ^3^Consejo Nacional de Investigaciones Científicas y Técnicas Buenos Aires, Argentina

**Keywords:** individual differences, factor analysis, five factor personality model, well-being, test validity

## Abstract

The study of individual differences in positive characteristics has mainly focused on moral traits. The objectives of this research were to study individual differences in positive characteristics from the point of view of the layperson, including non-moral individual characteristics, and to generate a replicable model of positive factors. Three studies based on a lexical approach were conducted. The first study generated a corpus of words which resulted in a refined list of socially shared positive characteristics. The second study produced a five-factor model of positive characteristics: erudition, peace, cheerfulness, honesty, and tenacity. The third study confirmed the model with a different sample. The five-positive-factor model not only showed positive associations with emotional, psychological and social well-being, but it also accounted for the variance beyond that accounted for by the Big Five factors in predicting these well-being dimensions. In addition, the presence of convergent and divergent validity of the five positive factors is shown with relation to the Values-in-Action (VIA) classification of character strengths proposed by Peterson and Seligman (2004).

## Introduction

Individual positive characteristics have been studied for thousands of years. The topic was considered by notable figures in Western and Eastern philosophies and religions, such as Confucius, Lao Tzu, Buddha, Plato, Aristotle, Moses, Solomon, Paul, Thomas Aquinas, Muhammad, and Al Farabi (Cosentino, 2010a). Since early times, this subject has also been of interest in Psychology. Studies on positive characteristics were published at the beginning (e.g., Thorndike, Hartshorne, May), middle (e.g., Erikson, Kohlberg, Maslow, Jahoda) and end (e.g., Buss, Ryff, Keyes) of the twentieth century (McCullough and Snyder, 2000; Peterson and Seligman, 2004).

Studies on positive individual characteristics have also been done in this century, and they may be classified as data-or theory-driven approaches. A significant example of a theory-driven approach is the classification of moral positive traits developed by Peterson and Seligman (2004). This remarkable classification originated in the study by Dahlsgaard et al. (2005), who proposed that six virtues were implicitly or explicitly mentioned in some traditional religious and philosophical texts from Eastern (e.g., Hinduism) and Western (e.g., Athenian philosophy) cultures. Based on a series of academic analysis and debates, these authors proposed 24 character strengths corresponding to those moral virtues.

The data-driven approach has produced different types of studies. Some authors use similarity ratings between concepts, while others use self-ratings on individual differences. An example of research from the similarity of concepts perspective is Walker and Pitts (1998) study on virtues. From the individual differences perspective, there are three well-known studies on moral positive characteristics. Cawley et al. (2000) identified 140 terms corresponding to virtues defined in a dictionary and asked university students to rate themselves on each of these terms. As a result, a four-dimensional model of virtues was proposed: empathy, order, resourcefulness, and serenity. In addition, De Raad and van Oudenhoven (2011), using a psycho-lexical approach, asked judges, psychologists and students to select 153 descriptors of virtues from a list of items previously drawn from adjectives listed in a dictionary. The participants, mostly students, were organized in pairs: each member self-rated him/herself and was rated by his/her partner on each of the virtue definitions. As a result, a six-factor model of virtues was proposed, consisting of sociability, achievement, respectfulness, vigor, altruism, and prudence. Finally, Morales-Vives et al. (2014) conducted a multi-step process that started with a list of thousands of words obtained from a database and identified 209 virtue definitions. Afterwards, university students rated themselves on each of these terms. As a result, a seven-factor model was obtained: self-confidence, rectitude, compassion, sociability, reflection, perseverance and effort, and serenity.

The aim of this study was to create a replicable model of factors comprising positive psychological human characteristics shared by a social group, based on the layperson's point of view. Our inductive research was inspired by the lexical approach used in individual differences studies. According to this approach, if an individual characteristic is important in the real world, for everyday social and practical interactions, people need to talk about it, and therefore, invent a word to name it (Goldberg, 1981). In particular, when it comes to judging other individuals, the ability to quickly determine to what extent people have certain general attributes (i.e., factors) is useful in anticipating future interactions and behaviors (McAdams, 1995).

Within this lexical hypothesis, a few basic but important replicable factors are selected, instead of a long list of idiosyncratic attributes with little chance of replication. This view is in agreement with several recent studies on personality (cf., Ashton and Lee, 2005; Saucier, 2009; De Raad et al., 2010).

To date, studies on individual positive characteristics have focused only on a subtype of positive traits: the moral ones (i.e., virtues and character strengths). Psychological talents, aptitudes, abilities or skills have been consistently excluded as they were deemed to be performance characteristics (e.g., Peterson and Seligman, 2004; De Raad and van Oudenhoven, 2011; cf., Saucier, 2009). Conversely, we consider that as these positive characteristics may be important in social interaction, the layperson may regard them as relevant traits. Consequently, our research aims to study positive psychological human characteristics in a broad sense, including non-moral (e.g., tranquility) as well as performance (e.g., intelligence) traits.

Although the inductive data-driven research presented above describes the layperson's point of view, contrary to theory-driven studies that develop upon ideas from academics and experts, we should note that inductive research also involves judges' or experts' participation in various instances, which specifically contributes to eliminating terms and generating a refined list of concepts or traits. Our approach attempted to be emphatically more data-driven, even to the extent that the inclusion or exclusion of words was done solely on the basis of the statistical analysis of frequency of word occurrence. We thus made efforts to prevent the idiosyncrasy of the researchers from having a bias effect on the definitive list of positive characteristics. Finally, we hypothesized that positive psychological human characteristics shared by a social group are a set of specific personality traits closely related to well-being. In this regard, some studies have shown the association of moral positive characteristics with personality and well-being (Peterson and Seligman, 2004; De Raad and van Oudenhoven, 2011; Cosentino and Castro Solano, 2015; Castro Solano and Cosentino, 2016).

## Study 1: development of a positive traits list

The aim of this study was to generate a list of positive psychological human characteristics shared by a social group. The selection of positive characteristics has traditionally been based on the intervention of experts and the inclusion of elements drawn from scholarly production. Such practice has been a source of concern for our inductive study, as these procedures could lead to distortion of or bias toward the point of view of the layperson. Therefore, to mitigate these concerns, two major decisions were made.

Firstly, although dictionaries have often been used as a starting point for lexical studies, an alternative procedure was used. This procedure consisted in creating a corpus of words from terms spontaneously generated by laypersons. We consider that using a corpus of words to develop a list of positive human characteristics involves some advantages: The items included in the corpus are words evidently used by laypersons, that is, they represent the popular and lively language; and the frequency of use of the word in plain language can be determined by the frequency of occurrence in the corpus of words, and thus, it may be used as a selection criterion. In addition, we assume that the words of the corpus are positive characteristics from the layperson's point of view, and that according to the instructions given to create the corpus of words (see Instrument subsection below), all the terms included are words commonly used to describe human beings.

Secondly, to generate a refined list of positive psychological human characteristics, the use of statistical and syntactical criteria (i.e., superficial or structural) was preferred to the semantic criteria of judges, juries or experts. The first criterion makes it possible not to exclude a priori from the list of positive qualities the terms with divergent syntaxes or structures that might be considered synonyms (cf., Rives, 2008; Rose, 2012).

### Sample

The convenience sample consisted of 745 participants (399 women) with a mean age of 37.7 (*SD* = 15.1). Participation was voluntary.

### Instrument

A qualitative questionnaire was developed to obtain words that represent positive psychological human characteristics. Participants were asked to think of a person they admired (not in terms of physical or economic characteristics) and to write down up to seven words that represented the personal qualities or characteristics that they admired the most.

### Procedure

The snowball sampling method was used for collecting the data. Undergraduate students of Psychology received academic credits for incorporating participants into the sample. These students were asked to select participants by using the following criteria: half of the participants should be over the age of 40, the other half under the age of 40; half of the participants should be female and the other half male. The participants did not receive any compensation for responding to the inventories. Participants responded to the instruments individually. An ethics statement is included below.

### Ethics statement

In all the studies, participation was voluntary and anonymous. The individuals gave their free and informed consent for their participation after being informed about the research objectives. They were also told that no explanation was required and that there would be no negative consequences if they decided to abandon the research at any time. An inclusion criterion was that participants should be 18 years of age or older. A formal evaluation by an ethics committee was not needed due to the research characteristics (voluntary and anonymous participation of adult individuals with informed consent in studies without intervention). However, the research met the ethics guidelines and it was tacitly approved by an in-house ethics committee at the Department of Psychology, Universidad de Palermo.

### Results

The participants' responses generated a list of 4,455 items corresponding to 854 words with different frequencies of occurrence. The 854 terms constituted the corpus of words of positive human characteristics. The 10 words with the highest frequency (see brackets) were “intelligent” (131), “intelligence” (120), “honesty” (91), “humility” (82), “kindness” (68), “persistent” (59), “hard-worker” (58), “humble” (56), “perseverance” (53), “humor” (47), and “strength” (44). A total of 406 words only presented a frequency of occurrence of 1.

In order to refine the corpus of words to a manageable list of positive psychological human characteristics, different procedures were used. Firstly, statistical and syntactical criteria were used to guide the reduction of the corpus. On the one hand, masculine and feminine adjectives of similar structure were unified in a single term, and the frequency of occurrence was considered as the sum of individual frequencies. For example, the items “*sincero*” (*n* = 17) and “*sincera*” (*n* = 32) were replaced by the item “*sincero/a*” (*n* = 49). (The word “*sincero*” in Spanish refers to a sincere male person and the word “*sincera*” refers to a sincere female person). In addition, words with the same root (i.e., lexeme or lexical base) were grouped in one item. The word with the highest frequency in the group was chosen as the representative item, and the sum of the frequencies of all items in the group was assigned as the frequency of the item. For example, for the group containing the items sincere (i.e., “*sincero/a*” in Spanish; *n* = 49) and sincerity (i.e., “*sinceridad*”; *n* = 36), the item sincere was chosen as the representative lexeme (49 > 36), with a corresponding frequency of 85 (i.e., 49 + 36). As a result of this procedure, the corpus of words was reduced to a list of 519 items, with a frequency ranging from 1 to 253.

Secondly, items with *n* = 1 were considered idiosyncratic, as there was no empirical evidence to consider them socially shared positive characteristics, and were therefore excluded from the list. Consequently, 301 representative words of socially shared positive psychological human characteristics were retained in the list, their frequencies presenting a median of 5 (52.5% cumulative percentage).

Thirdly, a more thorough refinement of the word list was made. To do this, we selected the items most frequently mentioned. Accordingly, 143 words with a frequency higher than the median (minimum frequency = 6) were selected. This list included items mentioned by <1% of the sample and items mentioned by 34% of the sample. In other words, the refined list included lowly-shared (low-frequency) and highly-shared (high-frequency) items among participants.

Finally, the 143 words were reviewed in order to select items representing positive psychological human characteristics that could be attributed to individuals beyond sex or age. Some items, such as “mother” (*n* = 15), “father” (*n* = 9), and “gentleman” (*n* = 7) presented problems in this regard and were therefore not considered for future analyses. The resulting number of items was 140 (see Instrument subsection below).

## Study 2: development of a positive factor model

The main objective of these analyses was to create a relatively pure and simple model of positive factors. We used a two-step procedure. Step 1 involved exploring the factors that derived from the data and then selecting those most clearly defined. Step 2 aimed at refining the factors selected in Step 1. Obtaining a refined model of positive features with few and pure markers would yield a measuring instrument of positive factors with a good fit to the data and short length. An instrument with such characteristics would be suitable for use in research conducted under severe time constraints and/or administered in conjunction with a number of other measurements, which is a common practice in psychological research.

### Sample

The convenience sample consisted of 1,000 people (571 women) with a mean age of 35.2 years (*SD* = 13.7, range 18–80). Participants were volunteers and did not receive any compensation. The sample data were divided randomly into two approximately equal parts. Subsample 1 (i.e., the sample for the model exploration) consisted of 516 participants (286 women) with a mean age of 35.2 (*SD* = 13.5, range 18–80). Subsample 2 (i.e., the sample for the model refinement) consisted of 484 participants (285 women) with a mean age of 35.1 (*SD* = 14.0, range 18–79).

### Procedure

Similarly to Study 1, participants were recruited by the snowball technique and they responded to the instruments individually.

### Step 1: model exploration

The first step aimed at finding a positive-factor model that could be replicated. Consequently, we only selected the factors that were clearly defined in the Exploratory Factor Analysis (EFA) outcome. The clearly defined factors were those that included at least four items per factor, with item-to-factor loadings considered good (i.e., loadings = 0.55), and at least one of these items should present an item-to-factor loading that could be classified as very good (loadings = 0.63), according to Comrey and Lee (1992) guidelines.

### Instrument

The resulting list of 140 items from Study 1, in decreasing order of frequency, was the starting point for developing an inventory of positive psychological characteristics. Participants were instructed to assess to what extent the items described them, using a scale ranging from 1 to 7. The higher the score, the greater the participants' identification with that feature. In order to help participants understand the task, a verb (usually to be or to have) was added before each item, indicating possession of the characteristic. For example, for the original item “intelligent,” the corresponding inventory item was “I'm intelligent” (see items in Table 1).

**Table 1:** Selected items in English (and in Argentinian Spanish).

1. **I'm intelligent (Soy inteligente)**
2. I'm honest (Tengo honestidad)
3. I'm humble (Tengo humildad)
4. I'm persevering (Soy perseverante)
5. I'm kind-hearted (Tengo bondad)
6. I'm generous (Soy generoso/a)
7. I'm hard-working (Soy trabajador/a)
8. I have strength (Tengo fortaleza)
9. I'm sincere (Soy sincero/a)
10. I'm a companion (Soy compañero/a)
11. I'm loving (Tengo amor)
12. I show solidarity (Soy solidario/a)
13. I'm brave (Soy valiente)
14. I'm creative (Tengo creatividad)
15. I'm patient (Tengo paciencia)
16. I'm cheerful (Soy alegre)
17. I'm affectionate (Soy cariñoso/a)
18. I'm responsible (Soy responsable)
19. I'm sensitive (Soy sensible)
20. I'm good (Soy bueno/a)
21. **I have humor (Tengo humor)**
22. I'm polite (Soy amable)
23. I'm a fighter (Soy luchador/a)
24. I'm understanding (Soy comprensivo/a)
25. I'm respectful (Soy respetuoso/a)
26. I'm faithful (Soy fiel)
27. **I'm wise (Tengo sabiduría)**
28. **I'm loyal (Tengo lealtad)**
29. **I'm pleasant (Soy simpático/a)**
30. **I'm amusing (Soy divertida/o)**
31. I'm friendly (Soy amigo/a)
32. I'm committed (Tengo compromiso)
33. I'm courageous (Tengo coraje)
34. I'm tenacious (Tengo tenacidad)
35. I'm charismatic (Soy carismático/a)
36. I'm capable (Soy capaz)
37. I'm a leader (Tengo liderazgo)
38. **I'm dedicated (Tengo dedicación)**
39. I'm optimistic (Soy optimista)
40. I'm modest (Tengo sencillez)
41. I have willpower (Tengo voluntad)
42. I have perseverance (Tengo constancia)
43. I'm enterprising (Soy emprendedor/a)
44. I have confidence (Tengo seguridad)
45. I'm positive (Soy positivo/a)
46. I'm fair (Soy justo/a)
47. I'm considerate (Soy atenta/o)
48. I'm audacious (Soy audaz)
49. I have integrity (Tengo integridad)
50. I'm simple (Tengo simpleza)
51. **I'm reliable (Soy confiable)**
52. I'm devoted (Tengo entrega)
53. I'm independent (Soy independiente)
54. I'm gifted (Tengo talento)
55. **I'm visionary (Soy visionario/a)**
56. I'm firm (Tengo firmeza)
57. I have personality (Tengo personalidad)
58. I make sacrifices (Tengo sacrificio)
59. **I'm tolerant (Soy tolerante)**
60. **I'm cultured (Soy culto/a)**
61. I'm selfless (Soy desinteresado/a)
62. I'm spiritual (Soy espiritual)
63. I have ability (Tengo habilidad)
64. I'm organized (Soy organizado/a)
65. I'm a protective (Soy protector/a)
66. **I have tranquility (Tengo tranquilidad)**
67. I'm altruistic (Tengo altruismo)
68. I'm genuine (Soy auténtico/a)
69. I'm decisive (Soy decidido/a)
70. I'm well-mannered (Soy educado)
71. I'm a thinker (Soy pensante)
72. **I'm persistent (Soy persistente)**
73. I'm sociable (Soy sociable)
74. I'm adventurous (Soy aventurero/a)
75. I have clarity (Tengo claridad)
76. I'm compassionate (Tengo compasión)
77. I'm confident (Tengo confianza)
78. I'm determined (Tengo determinación)
79. I'm sweet (Tengo dulzura)
80. I'm empathic (Soy empático/a)
81. I'm studious (Soy estudioso/a)
82. I have faith (Tengo fe)
83. **I'm funny (Soy gracioso/a)**
84. I'm helpful (Soy servicial)
85. **I have values (Tengo valores)**
86. **I have genius (Tengo genio)**
87. I'm coherent (Tengo coherencia)
88. I give advice (Soy consejero/a)
89. I'm a listener (Tengo escucha)
90. I'm spontaneous (Soy espontánea/o)
91. I'm humane (Tengo humanidad)
92. I'm intuitive (Soy intuitiva/o)
93. I'm original (Soy original)
94. **I have serenity (Tengo serenidad)**
95. **I'm transparent (Soy transparente)**
96. I have beauty (Tengo belleza)
97. I have personality (Tengo carácter)
98. I'm charitable (Soy caritativo/a)
99. **I'm a trier (Tengo esfuerzo)**
100. I'm successful (Soy exitoso/a)
101. I'm straightforward (Soy frontal)
102. **I'm ingenious (Tengo ingenio)**
103. I'm noble (Tengo nobleza)
104. I'm curious (Soy curioso/a)
105. I have energy (Tengo energía)
106. I have hope (Tengo esperanza)
107. I'm idealistic (Soy idealista)
108. I'm unassuming (Soy modesta/o)
109. I'm orderly (Soy ordenado/a)
110. I have passion (Tengo pasión)
111. I'm reflective (Soy reflexiva/o)
112. I'm seductive (Soy seductor/a)
113. I have attitude (Tengo actitud)
114. I'm warm (Soy cálido/a)
115. I'm consistent (Soy consecuente)
116. I'm supportive (Doy contención)
117. I'm amiable (Tengo cordialidad)
118. I'm home-loving (Soy familiero/a)
119. I'm honorable (Soy honorable)
120. I'm innovative (Soy innovador/a)
121. I have mercy (Tengo misericordia)
122. I'm a perfectionist (Soy perfeccionista)
123. I'm revolutionary (Soy revolucionaria/o)
124. I'm serious (Tengo seriedad)
125. I'm balanced (Tengo templanza)
126. I'm ambitious (Soy ambiciosa/o)
127. I'm shrewd (Tengo astucia)
128. I'm disciplined (Tengo disciplina)
129. I have endurance (Tengo entereza)
130. I'm a strategist (Soy estratega)
131. I'm an extrovert (Soy extrovertido/a)
132. I'm gentle (Soy gentil)
133. I'm grateful (Tengo gratitud)
134. I'm unconditional (Soy incondicional)
135. **I'm industrious (Tengo laboriosidad)**
136. I'm a speaker (Soy orador)
137. I'm resilient (Soy resiliente)
138. I'm a dreamer (Soy soñador/a)
139. **I'm truthful (Soy verdadero/a)**
140. I have vitality (Tengo vitalidad)

*This is a direct translation into English of the Argentinian Spanish items. Items selected for the final inventory are in boldface*.

### Results

The results of the exploratory step were as follows:

The data analyzed were polytomous ordered items because the scale to respond to items consisted of seven ordered categories. Consequently, it was not possible to obtain multivariate normality of the distribution of the sample. The MVN package (Version 4.0; Korkmaz et al., 2014) was used to analyse multivariate normality in the R environment for statistical computing (Version 3.2.4; R Core Team, 2016) with JGR (Helbig et al., 2013). Consistently, the results of Mardia's multivariate normality test revealed no normal multivariate data (Mardia's estimation of multivariate skewness = 7,395.67, skewness χ^2^ = 636,027.60, *p* < 0.001; Mardia's estimation of multivariate kurtosis = 22,051.87, kurtosis z = 123.71, *p* < 0.001) for the subsample 1.

The software FACTOR was used to determine the number of factors to be extracted (Version 10.4.01; Lorenzo-Seva and Ferrando, 2013). Given that the responses yielded 7 ordered categories and did not present a normal distribution, it was decided that the procedure for determining the number of dimensions should be parallel analysis based on minimum rank factor analysis (PA-MRFA; Timmerman and Lorenzo-Seva, 2011). This procedure included 500 random polychoric correlation matrices, obtained by permutation of the raw data. The result of PA-MRFA suggested eight factors to be retained.

Next, the factors were rotated to favor interpretability (Brown, 2006). As correlated dimensions are frequent in Psychology (Schmitt, 2011), and positive personal characteristic dimensions have been found to be associated (e.g., Singh and Choubisa, 2010), we selected the oblique rotation. From the several oblique rotation criteria for rotating the factors, we selected the Crawford-Ferguson (CF) Quartimax rotation because it produces cleaner factorial structures, similar to the Confirmatory Factor Analysis (CFA) results, a type of analysis that we used in a later step. This rotation had been used with data as ordered categories and it had produced interpretable factor solutions in our previous research (Cosentino and Castro Solano, 2014; Cosentino et al., in press). For the rotation procedure, we used the software Comprehensive Exploratory Factor Analysis (version 3.04; Tateneni et al., 2009). As a result of this rotation procedure with polychoric correlations and Kaiser row weights, we selected five factors because each of them satisfied the aforementioned criteria (i.e., including at least four items with good and very good item-to-factor loadings).

### Step 2: model refinement

The aim of the second step was to achieve a refined model, that is, a more definitive and much purer measure of the five positive factors so as to obtain a short measurement instrument with a good fit (Pett et al., 2003; Tabachnick and Fidell, 2013). To select the refined model of positive factors with pure markers, the following characteristics were deemed essential: a good fit to the data according to the indices Comparative Fit Index (CFI), Standardized Root Mean Square Residual (SRMR), and Root Mean Square Error of Approximation (RMSEA); the selected factors should consist of at least four items, and present a reliability higher than 0.80 (Kenny, 1979; Costello and Osborne, 2005). Consequently, we repeatedly explored several five-factor models defined with different markers until we obtained a refined model that met the criteria mentioned.

### Strategy for obtaining the refined model

A set of 57 items corresponding to the five positive factors was selected from the EFA result. Only items with at least fair item-to-factor loadings (0.45) in their factor were included. We used the subsample 2.

The objective was to reduce the number of items in the five-factor model selected from the EFA result, so that the factors would be in line with previously established criteria (i.e., good fit of the model to the data; omega reliability of each factor ≥0.80; and few items per factor, with a lower limit of 4 items). To refine the model, we used the structural equation modeling framed within an exploratory mode (Byrne, 2010). We proceeded in a manual and iterative way on successive structural equation modeling results to select a set of few items that were pure markers of the five factors, and thus obtain a refined model.

The model goodness of fit was evaluated by 3 indices CFI, SRMR, and RMSEA. Cut-off values close to and above 0.95 were considered for CFI, close to and lower 0.08 for SRMR, and <0.07 for RMSEA (Hu and Bentler, 1999; Steiger, 2007; Hooper et al., 2008; with the upper limit of confidence interval <0.08 as indicators of adequate adjustment). The R package lavaan was used for structural equation model analyses (Version 0.5–23.1097; Rosseel, 2012).

### Results

The refined model yielded the following factors: (markers in brackets): erudition (intelligent, wise, visionary, cultured, genius, ingenious; 6 items), peace (patient, tolerant, tranquility, serenity; 4 items), cheerfulness (humor, pleasant, funny, amusing; 4 items), honesty (loyal, reliable, values, transparent, truthful; 5 items), and tenacity (dedicated, persistent, effort, industrious; 4 items).

The result of the refined model was as follows: robust diagonally weighted least squares estimator (Robust DWLS) $\chi_{\left(220\right)}^{2}$ = 524.50, *p* < 0.05, CFI = 0.968, SRMR = 0.045, RMSEA = 0.054, (90% Confidence Interval = 0.048–0.059). This result was interpreted as a good fit to the data. The reliabilities of the factors tested with the coefficient omega for categorical items were higher than 0.80. Reliabilities were calculated with the semTools package (Version 0.4–11; semTools Contributors, 2016) in R (version 3.2.4; R Core Team, 2016). Consequently, the refinement step produced a model that satisfied all the previously described criteria.

## Study 3: confirmatory factor analysis and validity studies

This study aimed to confirm the model of factors consisting of relatively pure markers of positive psychological characteristics. A different sample from that of Study 2 was used. Additionally, it was hypothesized that factors of positive characteristics would be associated with the Big Five personality traits as well as with positive mental health, regarded as various forms of well-being. Because the relationships between positive factors and the Big Five are very important, the relationship between these models was studied. We assumed that none of the Big Five factors gives a single explanation for any of the positive factors, although some factors may be more closely associated than others. As the Big Five personality traits were found to be related to positive mental health (Lamers et al., 2012), we hypothesized that the positive factors would account for the variance of positive mental health above and beyond that accounted for by the personality factors. We also studied the convergent and divergent validity of the five positive factors using the Peterson and Seligman (2004) classification of 24 character strengths. We chose this classification because it includes many positive traits, some of which we assume to be similar to and others different from the five positive factors.

### Sample

The convenience sample consisted of 1,118 people (564 women) with a mean age of 40.4 years (*SD* = 14.2, range 18–92). Participants were volunteers who did not receive any contribution. Some participants additionally completed an instrument for assessing positive mental health. Therefore, the MHC subsample of 436 individuals (217 women; mean age = 40.9 years, *SD* = 14.9, range 18–92) was created. The other 682 participants (347 women) completed a measurement instrument of character strengths (mean age = 40.1 years, *SD* = 13.7, range 18–82), thus constituting the SCI subsample.

### Instruments

Four measurement instruments were used.

#### High five inventory (HFI)

The HFI is a measurement instrument composed of 23 items that correspond to relatively pure markers of five positive factors: erudition, peace, cheerfulness, honesty and tenacity (see Appendices 1 and 2 in Supplementary Materials). The factor erudition includes 6 items corresponding to the positive characteristics intelligent, wise, visionary, cultured, genius, and ingenious; the factor peace includes 4 items corresponding to patient, tolerant, tranquility, and serenity; the factor cheerfulness includes 4 items corresponding to humor, pleasant, funny, and amusing; the factor honesty includes 5 items corresponding to loyal, reliable, values, transparent, and truthful; and the factor tenacity includes 4 items corresponding to dedicated, persistent, effort, and industrious. The HFI was data-driven derived from the layperson point of view on positive characteristics. The model showed a good fit to data (see Step 2, Results subsection of Study 2). Omega coefficient for categorical items are shown in Table 2. Usual alpha coefficients were 0.82 for erudition; 0.84 for peace; 0.89 for cheerfulness; 0.84 for honesty; and 0.85 for tenacity.

**Table 2:** Correlations between positive psychological factors and a series of relevant variables.

**Scale**	**Erudition**	**Peace**	**Cheerfulness**	**Honesty**	**Tenacity**
HFI Erudition	(0.84)				
HFI Peace	[0.46] 0.36^***^	(0.86)			
HFI Cheerfulness	[0.59] 0.44^***^	[0.45] 0.31^***^	(0.88)		
HFI Honesty	[0.46] 0.45^***^	[0.43] 0.35^***^	[0.41] 0.34^***^	(0.88)	
HFI Tenacity	[0.54] 0.54^***^	[0.41] 0.33^***^	[0.30] 0.26^***^	[0.71] 0.60^***^	(0.86)
MHC-SF Emotional	0.24^***^	0.25^***^	0.31^***^	0.23^***^	0.29^***^
MHC-SF Social	0.26^***^	0.28^***^	0.22^***^	0.16^***^	0.24^***^
MHC-SF Psychological	0.38^***^	0.29^***^	0.35^***^	0.33^***^	0.38^***^
BFI Extraversion	0.24^***^	0.02	0.46^***^	0.15^***^	0.19^***^
BFI Agreeableness	0.13^***^	0.40^***^	0.25^***^	0.37^***^	0.27^***^
BFI Conscientiousness	0.27^***^	0.18^***^	0.04	0.25^***^	0.52^***^
BFI Neuroticism	−0.17^***^	−0.45^***^	−0.23^***^	−0.14^***^	−0.10^***^
BFI Openness	0.38^***^	0.14^***^	0.21^***^	0.16^***^	0.15^***^
Assertiveness (Extraversion)	0.16^***^	−0.07^*^	0.37^***^	0.06	0.07^*^
Activity (Extraversion)	0.31^***^	0.15^***^	0.37^***^	0.18^***^	0.34^***^
Altruism (Agreeableness)	0.15^***^	0.27^***^	0.26^***^	0.36^***^	0.26^***^
Compliance (Agreeableness)	0.05	0.35^***^	0.14^***^	0.26^***^	0.16^***^
Order (Con.)	0.13^***^	0.10^**^	0.02	0.15^***^	0.30^***^
Self-Discipline (Con.)	0.26^***^	0.18^***^	0.02	0.25^***^	0.52^***^
Anxiety (Neuroticism)	−0.16^***^	−0.39^***^	−0.20^***^	−0.10^***^	−0.04
Depression (Neuroticism)	−0.13^***^	−0.30^***^	−0.21^***^	−0.12^***^	−0.12^***^
Esthetics (Openness)	0.20^***^	0.09^**^	0.10^***^	0.08^*^	0.03
Ideas (Openness)	0.38^***^	0.12^***^	0.22^***^	0.18^***^	0.17^***^
SCI Appreciation	0.09^*^	0.11^**^	0.09^*^	0.11^**^	0.06
SCI Fairness	−0.04	0.15^***^	0.07	0.19^***^	0.11^**^
SCI Persistence	0.16^***^	0.12^**^	0.01	0.15^***^	0.40^***^
SCI Creativity	0.27^***^	0.08^*^	0.17^***^	0.08^*^	0.15^***^
SCI Love	0.09^*^	0.03	0.21^***^	0.22^***^	0.15^***^
SCI Self-regulation	0.07^†^	0.15^***^	−0.03	0.03	0.16^***^
SCI Gratitude	0.19^***^	0.12^**^	0.11^**^	0.16^***^	0.19^***^
SCI Leadership	0.19^***^	0.09^*^	0.16^***^	0.17^***^	0.13^**^
SCI Judgment	0.04	0.07^†^	−0.10^**^	0.12^**^	0.18^***^
SCI Social Intelligence	0.15^***^	0.13^***^	0.25^***^	0.20^***^	0.17^***^
SCI Forgiveness	0.07	0.26^***^	0.08^*^	0.19^***^	0.14^***^
SCI Spirituality	0.10^**^	0.12^**^	0.06	0.17^***^	0.19^***^
SCI Teamwork	0.03	0.07^†^	0.17^***^	0.15^***^	0.07
SCI Bravery	0.18^***^	−0.07	0.09^*^	0.09^*^	0.09^*^
SCI Curiosity	0.28^***^	0.13^**^	0.15^***^	0.21^***^	0.24^***^
SCI Kindness	0.06	0.16^***^	0.20^***^	0.28^***^	0.17^***^
SCI Hope	0.21^***^	0.20^***^	0.14^***^	0.18^***^	0.22^***^
SCI Integrity	0.08^†^	0.20^***^	0.07	0.31^***^	0.24^***^
SCI Perspective	0.19^***^	0.23^***^	0.07	0.19^***^	0.21^***^
SCI Prudence	0.05	0.19^***^	−0.05	0.25^***^	0.24^***^
SCI Humor	0.14^***^	00.12^**^	0.47^***^	0.17^***^	0.04
SCI Humility	−0.11^**^	0.20^***^	−0.11^**^	0.18^***^	0.11^**^
SCI Love of learning	0.10^**^	0.04	−0.04	0.05	0.09^*^
SCI Zest	0.18^***^	0.15^***^	0.12^**^	0.12^**^	0.27^***^

*HFI, High Five Inventory; MHC, The Mental Health Continuum-Short Form; BFI, Big Five Inventory; SCI, Strengths of Character Inventory; Con., Conscientiousness. The values in parentheses are scale ω reliabilities. The values in square brackets are latent variable intercorrelations from Study 2*.

*N = 1,118, except correlations with MHC (n = 436), and correlations with SCI (n = 682)*.

* *p < 0.05*.

** *p < 0.01*.

*** *p < 0.001*.

† *p < 0.07*.

#### Big five inventory (BFI)

A locally adapted version of the BFI (Castro Solano and Casullo, 2001) was used to assess the Big Five model of personality: extraversion, agreeableness, conscientiousness, neuroticism, and openness (John et al., 1991). This instrument consists of 44 items with statements about personality characteristics that are answered on a 5-point Likert scale. The factorial structure of the adapted BFI version was verified with different samples (Castro Solano, 2005). The omega coefficient for categorical items (alpha internal consistency in square brackets) were 0.69 [0.75] for extraversion; 0.70 [0.75] for neuroticism; 0.73 [0.71] for agreeableness; 0.81 [0.80] for conscientiousness and 0.81 [0.79] for openness. The BFI version used has allowed us to make a decomposition into facet scales (Soto and John, 2009). Facet omega reliabilities for this sample [alpha in square brackets]: 0.75 [0.67] for assertiveness (extraversion); 0.64 [0.63] for activity (extraversion); 0.65 [0.51] for altruism (agreeableness); 0.48 [0.45] for compliance (agreeableness); 0.68 [0.66] for order (conscientiousness); 0.77 [0.69] for self-discipline (conscientiousness); 0.55 [0.58] for anxiety (neuroticism); 0.54 [0.51] for depression (neuroticism); for 0.72 [0.67] Esthetics (openness); and 0.70 [0.62] for ideas (openness).

#### Mental health continuum-short form (MHC-SF)

This instrument, developed by Keyes (2005), evaluates positive mental health composed of three types of well-being from the hedonic and eudemonic traditions: emotional, psychological and social well-being (Lamers et al., 2012). The adaptation of the MHC-SF used is psychometrically reliable and valid (Lupano Perugini et al., 2017). This instrument consists of 14 items that assess how participants have felt over the last month. Responses are given on a 6-point Likert scale. The omega for categorical items for this sample (alpha in square brackets) and the subscales were 0.78 [0.77] for emotional; 0.85 [0.77] for social; and 0.86 [0.84] for psychological well-being.

#### Strengths of character inventory (SCI)

This measure, developed by Cosentino and Castro Solano (unpublished manuscript), evaluates the 24 strengths of character from the Peterson and Seligman (2004) classification. This inventory includes 24 bipolar items (corresponding to the 24 character strengths), but enhancing the content validity of each item. The SCI asks respondents to indicate to what degree they identify with one of two self-descriptions: one that describes a character strength and the other that lacks the same character strength. The score for each item ranges from 1 (*I am very similar to the first person*) to 5 (*I am very similar to the second person*). Half of the items are reverse scored. The greater the score, the greater the presence of the character strength. The SCI is associated with measures of life satisfaction and the Big Five (Cosentino and Castro Solano, 2015). Besides, associations with more than medium effect size were found between self-rating and observer's rating. The SCI has an acceptable test-retest reliability, with *r*s in the range of 0.72–0.92, *M* = 0.80 (Cosentino, 2010b). The test-retest correlations are particularly important because internal consistency indices are not calculated for the SCI as each character strength is measured with a single item (Gosling et al., 2003). However, the omega and alpha may be calculated on the scores of the 24 items, these coefficients being considered indicators of the degree to which the participants' responses to the items are interrelated (i.e., they co-vary; Helms et al., 2006). For this sample, the omega total scale was 0.85 and the Cronbach alpha was 0.83.

### Procedure

The procedure was the same as that used in Studies 1 and 2 (i.e., the participants were recruited by the snowball technique, and they individually responded to the instruments).

### Results

#### CFA

As data consisted of polytomous ordered items, multivariate normality of the distribution of the sample could not be obtained. Consistently, results of Mardia's Multivariate Normality Test revealed that the data were not multivariate normal (Mardia's estimation of multivariate skewness = 51.67, skewness χ^2^ = 9,627.57, *p* < 0.001; Mardia's estimation of multivariate kurtosis = 785.69, kurtosis z = 103.87, *p* < 0.001).

The positive factors model with 23 indicators showed a good fit to data: Robust DWLS $\chi_{\left(220\right)}^{2}$ = 1,213.25, *p* < 0.05, CFI = 0.963, SRMR = 0.043, RMSEA = 0.064 (90% Confidence Interval = 0.060–0.067).

#### Associations of HFI with relevant variables

Table 2 shows Pearson's product-moment correlations among the HFI, the MHC, and the BFI scales. The HFI linear intercorrelations were all positive and statistically significant, *r*s range 0.26–0.60. All linear correlations among the HFI and the MHC were positive and statistically significant, *r*s range 0.16–0.38. The linear correlations among the HFI and the BFI factors were statistically significant, range *r*s |0.10|–|0.52|. However, the positive factors peace and extraversion, and the positive factors cheerfulness and conscientiousness were not found to be statistically significantly associated, respectively. Linear correlations among the HFI and the BFI factors were all positive, except for the neuroticism factor, which showed a negative association. The linear correlations among the HFI and the BFI facets were statistically significant, range *r*s |0.07|–|0.52|. However, six BFI facets were not found to be statistically associated with the positive factors. Linear correlations among the HFI and the BFI facets were all positive, except for the associations between the HFI positive factors and depression and anxiety (neuroticism facets), which showed a negative association. Another exception was the correlation between the positive factors peace and assertiveness (extraversion facet) which showed a negative association.

#### Regression between the big five and the positive factor model

A stepwise regression was performed in both directions to determine whether more than one Big Five factor could explain each positive factor. In this way, there would be no empirical support for the strong idea that the positive factors are merely a positive reflection of the Big Five. The results showed that the five Big Five factors (in brackets, factors with statistically significant beta coefficients, *p* < 0.05, in decreasing order) account for the variance in erudition (openness, 0.33, conscientiousness, 0.20, neuroticism, −0.09, extraversion, 0.08, and agreeableness, −0.07; *R*^2^ = 0.20); four factors account for the variance in peace (neuroticism, −0.36, agreeableness, 0.28, extraversion, −0.13, and openness, 0.06; 0.28) and in cheerfulness (extraversion, 0.44, conscientiousness, −0.17, agreeableness, 0.16, and neuroticism, −0.14; 0.27); three factors account for the variance in tenacity (conscientiousness, 0.50, agreeableness, 0.13, neuroticism, 0.08; 0.29); and two factors account for the variance in honesty (agreeableness, 0.32, and conscientiousness, 0.14; 0.15).

In other words, the results show that the hypothesis that the five positive factors are a simple positive reflection of the Big Five factors cannot be confirmed because no single personality factor accounts for all the variance for any of the positive factors. The most obvious case in point is the positive factor peace: neuroticism and agreeableness present weights relatively close to each other in accounting for the variance of the positive factor.

#### Convergent and discriminant validity

Analysis of the correlations between the five positive factors and the character strengths showed that they converged as follows: the cheerfulness factor and the character strengths humor, tenacity and persistence, honesty and integrity, with an effect size between medium and large, respectively [using Cohen (1992); see Table 2]. With an effect size between small and medium, the peace factor showed associations with forgiveness and perspective; and the erudition factor showed associations with creativity and curiosity. All positive factors showed absence of statistically significant associations, or showed effect sizes less than small with some character strengths, which supports their divergent validity. For example, no associations were found between the following factors and character strengths: erudition and teamwork, peace and love, cheerfulness and persistence, honesty and self-regulation, and tenacity and humor.

To complement the association analyses, a stepwise regression was performed in both directions to determine more precisely which character strengths of the Peterson and Seligman (2004) classification could account for the variance of the five positive factors. In what follows, the positive factors are ordered in decreasing order, according to the magnitude of the first beta. The variance of the positive factor cheerfulness were accounted for by the character strengths humor, judgment, love, social intelligence, prudence, and creativity (0.41, −0.13, 0.13, 0.11, −0.08, 0.07, statistically significant beta coefficients, *p* < 0.05, in decreasing order, respectively; R-squared = 0.28); the positive factor tenacity by the character strengths persistence, prudence, zest, curiosity, and spirituality (0.29, 0.11, 0.10, 0.10, 0.08; 0.21); the positive factor erudition by the character strengths curiosity, creativity, and humility (0.22, 0.19, −0.10; 0.13); the positive factor peace by the character strengths forgiveness, perspective, humility, bravery, hope, self–regulation, and humor (0.18, 0.15, 0.14, −0.10, 0.10, 0.10, 0.09; 0.16); and the positive factor honesty by the character strengths integrity, kindness, love, prudence, humility, and curiosity (0.17, 0.15, 0.10, 0.09, 0.09, 0.09; 0.17).

As expected, the results of the stepwise regression are consistent with those of the associations between positive factors and character strengths. Also, it should be noted that the participation of the character strengths is consistent with the positive factors it accounts for. However, while the positive factors cheerfulness and tenacity seem to be clearly accounted for mainly by a single character strength (humor and persistence, respectively), such clarity is lost in the accountability of erudition, peace, and honesty: not only are their first weights much lower, but also their highest weights do not differ much from their second highest weights (and for honesty, these in turn do not differ much from their third highest weights).

#### Hierarchical regression analysis

Six hierarchical multiple regression analyses were conducted to test the hypothesis that the positive factors can additionally account for the variance of the three types of well-being corresponding to positive mental health, beyond the variance accounted for by personality factors and facets, respectively. Consequently, personality factors (or facets) were entered in the first block of the equation, and the positive factors in the second block. Model comparison showed that positive variables increased the capacity to explain emotional well-being, psychological well-being, and social well-being variances (see Tables 3, 4).

**Table 3:** BFI factor and HFI factor standardized regression coefficients in a hierarchical regression analysis on positive mental health.

	**Predictor**	**Model 1**	**Model 2**
**EMOTIONAL WELL-BEING**			
	Extraversion	0.11^*^	0.01
	Agreeableness	0.07	0.01
	Conscientiousness	0.12^*^	0.05
	Neuroticism	−0.28^***^	−0.29^***^
	Openness	0.09^†^	0.07
	Erudition		−0.03
	Peace		−0.05
	Cheerfulness		0.20^***^
	Honesty		0.05
	Tenacity		0.18^**^
*R*^2^		0.17	0.23
*F*		18.20^***^	12.92^***^
Δ*R*^2^			0.0585
Model 2 v.1, *F*_(5, 425)_			6.49^***^
**PSYCHOLOGICAL WELL-BEING**			
	Extraversion	0.15^***^	0.04
	Agreeableness	0.14^**^	0.08
	Conscientiousness	0.22^***^	0.15^**^
	Neuroticism	−0.12^**^	−0.12^*^
	Openness	0.10^*^	0.05
	Erudition		0.11^†^
	Peace		−0.02
	Cheerfulness		0.19^***^
	Honesty		0.10^†^
	Tenacity		0.10
*R*^2^		0.20	0.29
*F*		21.79^***^	17.52^***^
Δ*R*^2^			0.0896
Model 2 v.1, *F*_(5, 425)_			10.76^***^
**SOCIAL WELL-BEING**			
	Extraversion	0.13^**^	0.11^*^
	Agreeableness	0.04	−0.01
	Conscientiousness	0.16^**^	0.11^†^
	Neuroticism	−0.17^***^	−0.11^*^
	Openness	0.06	0.03
	Erudition		0.07
	Peace		0.15^*^
	Cheerfulness		0.05
	Honesty		−0.02
	Tenacity		0.06
*R*^2^		0.12	0.16
*F*		11.54^***^	7.89^***^
Δ*R*^2^			0.0382
Model 2 v.1, *F*_(5, 425)_			3.85^**^

* *p < 0.05*.

** *p < 0.01*.

*** *p < 0.001*.

† *p < 0.07*.

**Table 4:** BFI facet and HFI factor standardized regression coefficients in a hierarchical regression analysis on positive mental health.

	**Predictor**	**Model 1**	**Model 2**
**EMOTIONAL W-B**			
	Assertiveness (Extraversion)	−0.04	−0.07
	Activity (Extraversion)	0.27^***^	0.20^***^
	Altruism (Agreeableness)	0.05	0.01
	Compliance (Agreeableness)	0.02	0.01
	Order (Con.)	−0.03	−0.03
	Self-Discipline (Con.)	0.07	0.04
	Anxiety (Neuroticism)	−0.15^**^	−0.16^**^
	Depression (Neuroticism)	−0.17^**^	−0.15^**^
	Esthetics (Openness)	0.00	0.01
	Ideas (Openness)	0.04	0.03
	Erudition		−0.02
	Peace		−0.03
	Cheerfulness		0.15^**^
	Honesty		0.08
	Tenacity		0.09
*R*^2^		0.22	0.25
*F*		11.87^***^	9.40^***^
Δ*R*^2^			0.0331
Model 2 v.1, *F*_(5, 420)_			3.71^**^
**PSYCHOLOGICAL W-B**			
	Assertiveness (Extraversion)	−0.01	−0.05
	Activity (Extraversion)	0.29^***^	0.21^***^
	Altruism (Agreeableness)	0.16^**^	0.11^*^
	Compliance (Agreeableness)	−0.02	−0.04
	Order (Con.)	0.02	−0.01
	Self-Discipline (Con.)	0.14^**^	0.12^*^
	Anxiety (Neuroticism)	−0.11^*^	−0.11^*^
	Depression (Neuroticism)	−0.05	−0.02
	Esthetics (Openness)	0.01	0.01
	Ideas (Openness)	0.02	−0.03
	Erudition		0.14^*^
	Peace		−0.02
	Cheerfulness		0.14^**^
	Honesty		0.14^**^
	Tenacity		0.02
*R*^2^		0.24	0.31
*F*		13.32^***^	12.82^***^
Δ*R*^2^	0.0754		
Model 2 v.1, *F*_(5, 420)_			9.24^***^
**SOCIAL W-B**			
	Assertiveness (Extraversion)	0.05	0.06
	Activity (Extraversion)	0.18^***^	0.12^***^
	Altruism (Agreeableness)	0.02	0.00
	Compliance (Agreeableness)	0.04	0.02
	Order (Con.)	0.02	0.01
	Self-Discipline (Con.)	0.11^*^	0.10
	Anxiety (Neuroticism)	−0.16^**^	−0.12^*^
	Depression (Neuroticism)	0.01	0.02
	Esthetics (Openness)	−0.01	−0.02
	Ideas (Openness)	0.01	−0.01
	Erudition		0.08
	Peace		0.14^**^
	Cheerfulness		0.04
	Honesty		0.00
	Tenacity		0.01
*R*^2^		0.13	0.16
*F*		6.19^***^	5.36^***^
Δ*R*^2^			0.0335
Model 2 v.1, *F*_(5, 420)_			3.36^**^

*W-B, Well-being; Con., Conscientiousness*.

* *p < 0.05*.

** *p < 0.01*.

*** *p < 0.001*.

## Discussion

This study shows a new strategy to obtain a model of individual characteristics using a lexical approach in the study of individual differences. This procedure involved developing a corpus of words and its refinement by applying statistical and syntactical procedures, which was useful in obtaining factors of positive psychological human characteristics from the point of view of the layperson. Additionally, unlike other studies on positive characteristics that have focused only on moral characteristics, this study included positive, non-moral traits. As a result, the analysis of socially shared positive psychological human characteristics generated a model of five inductively-derived positive factors: erudition, peace, cheerfulness, honesty and tenacity. The replication study with a different sample provides evidence for the construct validity of the new model. The positive relationship found between each positive factor with positive mental health, beyond normal personality, contributes soundness and validity to the new model. We call this inductively-derived five-positive-factor model the High Five model.

The High Five model consists of five positive psychological traits that we call *high factors* or simply *high*. As positive psychological traits, the high factors are present in each individual in a relatively stable way, and they are represented by positive psychological characteristics. The high factors have certain attributes: they can be measured, they vary across individuals, and they could putatively be increased or decreased by internal and/or external influences. The laypersons' concepts about the positive psychological characteristics of individuals were used as a basis for determining the high factors and their positive characteristics. Therefore, we assume that the positive psychological traits of the High Five model are particularly relevant to the layperson because individuals use them in their daily lives. Thus, when an individual determines the level of each of the high factors for a particular person, he or she may figure the type of social interaction that he or she may engage in with this person.

Our markedly inductive procedure has yielded five positive factors that were found to be related to the Big Five. In general terms, these positive factors could be considered to represent the positive poles of the Big Five. The strong hypothesis states that there is an exclusive and biunivocal relationship between the five positive factors and the Big Five, while the weak hypothesis states that each positive factor relates mainly to a Big Five factors. The associations between the Big Five and their regressions on the positive factors suggest that the relationship between the models is never exclusive and biunivocal. Instead, each dimension of the personality is associated mainly with a positive factor. Thus, the positive factor erudition is associated with the personality factor openness, peace with emotional stability (as opposed to neuroticism), cheerfulness with extraversion, honesty with agreeableness, and tenacity with conscientiousness. However, we believe that a more accurate characterization of the positive factors is achieved in light of the Big Five when we include the other personality factors, and not only those that mainly account for the variances of the positive factors. This is especially so for the positive factor peace and its relations with personality factors neuroticism and agreeableness. Therefore, a weak version of the hypothesis that positive factors represent the positive poles of the Big Five could be supported.

The results showed that each of the positive factors has positive associations with the different types of well-being of the positive mental health model. This relationship might be explained by the fact that the five positive factors represent thoughts, emotions, and behaviors that belong to both, intimate and social interactions, leading to people's hedonic, personal and social goals. In addition, the inductively-derived five-positive-factor model can account for the variance of these different types of well-being, above the variance accounted for by the Big Five model. In short, this new model not only accounts for different types of well-being, but also contributes to them beyond the influence of personality. This incremental validity is an indicator of the construct validity of the inductively-derived five-positive-factor model.

The results of associations and regressions of character strengths with positive factors are sufficient evidence to show their convergence and divergence on the classification of Peterson and Seligman (2004). The positive factors converge on constructs that are similar and diverge on those that do not present conceptual similarities.

In particular, we believe that the results of Big Five regressions and of the character strengths regressions on the positive factors, respectively, are useful in determining the nomologic network of each positive factor in the space of normal personality and of character strengths. In none of these spaces is the coincidence complete, although they are satisfactorily linked to conceptually close constructs. Consequently, we assume that the positive factors are constructs that differ from the personality according to the Big Five and the character strengths of the Peterson and Seligman (2004) classification.

### Limitations

All the samples of participants used in this research were convenience samples and, therefore, cannot guarantee the representativeness of the population. Although the BFI adaptation we used in this study had not been validated for its facets but only for its dimensions, the facet scores were calculated, and analyses with the BFI facets were included in this research. Besides, the BFI facets showed low or just-enough reliability. Consequently, the results of the BFI facets should be interpreted in light of these particular aspects. A major weakness of this research is that it was not possible to rely on culturally adapted measurement instruments, such as those used in inductive studies of the positive moral characteristics of Cawley et al. (2000), De Raad and van Oudenhoven (2011), and Morales-Vives et al. (2014), which have been very important antecedents to this research. As a result, it has not been possible to make the HFI validity studies with these models. Finally, the cross–sectional design of the study is another major limitation, since longitudinal studies could provide relevant information on the characteristics of HFI.

### Merits and future lines of research

For the first time, a study marked by a deliberately inductive methodology has the potential to generate a consistent positive-factor model composed of the positive characteristics proposed by laypersons. The positive-factor model shows its potential in explaining positive mental health beyond the Big Five contribution to it. In addition, our model differs from the classification of 24 character strengths (Peterson and Seligman, 2004). However, future research should study the empirical relationships between the five positive factors and the models of Cawley et al. (2000), De Raad and van Oudenhoven (2011), and Morales-Vives et al. (2014). In addition, future studies could replicate the HFI model in populations of other ages (children, adolescents, and the elderly) as well as in other countries and languages. Longitudinal studies may also be useful in determining whether the High Five model contributes to well-being over time and in defining significant psychometric properties of the HFI, such as the test-retest stability and the measurement invariance across time.

## Concluding remarks

People use words to communicate with each other, and they use certain words on a daily basis to describe positive characteristics of human beings. We have found that the positive characteristics proposed by laypersons are clearly and consistently linked to each other, in such a way that they delimit positive personal factors. We now have a clear and concise model of the five positive traits of personality arising from the ideas that laypersons have about what is admirable in human beings. We emphasize that the five-positive-factor model arose not from theoretical conceptions in the minds of philosophers, religious thinkers, moralists, or scholars, but inductively from the mindset of laypersons.

Although this novel study is based on a model that has not been supported by successive investigations yet, we wish to include some final comments, which should be taken with caution given their speculative nature. We therefore assume that this research provides suggestions and orientations regarding the psychology of the layperson, for whom our interventions and professional help are meant. On the one hand, it would be possible to assume with whom laypersons would like to share their social relations in different areas of their lives. It would not be surprising to find that people would feel comfortable sharing the different activities of their lives with people who have high erudition, high peace, high cheerfulness, high honesty, high tenacity. On the other hand, what better way to help, and intervene on our clients than to strengthen and develop their five positive traits? In addition, as these traits arose from the minds of laypersons, they may contribute to clients' identification and commitment to personal development. Ultimately, cultivating their High Five will lead them to improve their positive mental health, that is, to enhance their emotional, psychological and social well-being.

## Author contributions

AC and ACS provided substantial contributions to the conception and design of the work; the acquisition, analysis, and interpretation of data; drafting the work and revising it critically for important intellectual content; the final approval of the version to be published; and agreement to be accountable for all aspects of the work in ensuring that questions related to the accuracy or integrity of any part of the work are appropriately investigated and resolved.

### Conflict of interest statement

The authors declare that the research was conducted in the absence of any commercial or financial relationships that could be construed as a potential conflict of interest.
