# Supplementary material for: The High Five: Associations of the Five Positive Factors with the Big Five and Well-being
Source: Front Psychol. 2017 Jul 25;8:1250. doi: 10.3389/fpsyg.2017.01250 (PMC5524674; doi:10.3389/fpsyg.2017.01250)
Supplement: Supplementary file 1 [file DataSheet1.docx]

Appendix 1. High Five Inventory in Argentinian Spanish

HFI

Instrucciones: Abajo aparece una serie de afirmaciones que las personas utilizan para describirse a sí mismas. Leé cada afirmación y elegí la respuesta apropiada para indicar cómo vos te sentís generalmente utilizando la escala que aparece abajo. No hay respuestas correctas ni incorrectas. No pases mucho tiempo con cada afirmación, pero da la respuesta que te parece que describe cómo te sentís generalmente.

nunca casi nunca a veces a menudo muy seguido casi siempre siempre

1 2 3 4 5 6 7

Por favor, ubicá los números correspondientes a tus respuestas en la columna "frec." (de "frecuencia")

frec. afirmación frec. afirmación

soy inteligente soy divertido/a

tengo paciencia tengo valores

tengo humor tengo esfuerzo

tengo lealtad tengo genio

tengo dedicación tengo serenidad

tengo sabiduría soy gracioso/a

soy tolerante soy transparente

soy simpático/a tengo laboriosidad

soy confiable soy culto/a

soy persistente soy verdadero/a

soy visionario/a tengo ingenio

tengo tranquilidad

Appendix 2. High Five Inventory in English

HFI

Instructions: Listed below are a series of statements that people use to describe themselves. Read each statement and choose an appropriate answer using the scale below to indicate how you usually feel. There are no right or wrong answers. Do not spend a lot of time on each statement.

Never almost never sometimes often very often almost always always

1 2 3 4 5 6 7

Please, place the numbers corresponding to your answers in the column "freq." (for frequency")

freq. Statement freq. Statement

I´m intelligent I´m amusing

I´m patient I have values

I have humour I’m a trier

I´m loyal I have genius

I´m dedicated I have serenity

I´m wise I´m funny

I´m tolerant I´m transparent

I´m pleasant I´m industrious

I´m reliable I´m cultured

I´m persistent I´m truthful

I´m visionary I´m ingenious

I have tranquillity

*Note*. This is a direct adaptation and translation into English.
